# Transfontanellar shear wave elastography of the neonatal brain for quantitative evaluation of white matter damage

**DOI:** 10.1038/s41598-024-60968-w

**Published:** 2024-05-23

**Authors:** Flora Faure, Marianne Alison, Mariantonietta Francavilla, Priscilla Boizeau, Sophie Guilmin Crepon, Chung Lim, Gregory Planchette, Mickael Prigent, Alice Frérot, Mickael Tanter, Charlie Demené, Olivier Baud, Valérie Biran

**Affiliations:** 1 Institute Physics for Medicine Paris, Inserm U1273, ESPCI Paris, CNRS UMR 8063, PSL University, 75015 Paris, France; 2 Assistance Publique-Hôpitaux de Paris, Pediatric Radiology Department, Robert Debré University Hospital, 75019 Paris, France; 3 Pediatric Radiology Department, A.O.U.C. Policlinico of Bari - Hospital Giovanni XXIII, Bari, Italy; 4 Assistance Publique-Hôpitaux de Paris, Unit of Clinical Epidemiology, Inserm U1123 and CIC-EC 1426, Robert Debré Children’s Hospital, University of Paris Cité, Paris, France; 5 Department of Neonatal Intensive Care Unit, Assistance Publique-Hôpitaux de Paris, Robert Debré Children’s Hospital, Paris, France; 6 Division of Neonatology and Paediatric Intensive Care, Children’s University Hospital of Geneva and University of Geneva, Geneva, Switzerland; 7 Inserm U1141, University of Paris Cité, Paris, France

**Keywords:** Neonatal brain damage, Preterm birth

## Abstract

Cerebral white matter damage (WMD) is the most frequent brain lesion observed in infants surviving premature birth. Qualitative B-mode cranial ultrasound (cUS) is widely used to assess brain integrity at bedside. Its limitations include lower discriminatory power to predict long-term outcomes compared to magnetic resonance imaging (MRI). Shear wave elastography (SWE), a promising ultrasound imaging modality, might improve this limitation by detecting quantitative differences in tissue stiffness. The study enrolled 90 neonates (52% female, mean gestational age = 30.1 $\pm \hspace{0.17em}$4.5 weeks), including 78 preterm and 12 term controls. Preterm neonates underwent B-mode and SWE assessments in frontal white matter (WM), parietal WM, and thalami on day of life (DOL) 3, DOL8, DOL21, 40 weeks, and MRI at term equivalent age (TEA). Term infants were assessed on DOL3 only. Our data revealed that brain stiffness increased with gestational age in preterm infants but remained lower at TEA compared to the control group. In the frontal WM, elasticity values were lower in preterm infants with WMD detected on B-mode or MRI at TEA and show a good predictive value at DOL3. Thus, brain stiffness measurement using SWE could be a useful screening method for early identification of preterm infants at high WMD risk.

**Registration numbers**: EudraCT number ID-RCB: 2012-A01530-43, ClinicalTrial.gov number NCT02042716.

## Introduction

Prematurity is a global health problem and the leading cause of death in children under 5 years of age^1,2^. Despite major improvements in clinical care during the perinatal period in recent decades^3^, preterm infants remain a population at high risk of developing postnatal adverse events associated with brain injury, in particular white matter damage (WMD), that can lead to lifelong neurodevelopmental disorders^4^. WMD are secondary to ischemic or hemorrhagic injuries such as venous infarct. Ischemic injuries of white matter include focal cystic, non-cystic and diffuse periventricular leukomalacia^5^. Transfontanellar cranial ultrasound (cUS) is routinely used in neonatal intensive care units for early screening of brain injuries of preterm neonates, particularly those born before 32 weeks’ gestation^6^. B-mode imaging offers gray-scale anatomical images of the brain enabling to detect morphological abnormalities, ischemic or hemorrhagic injuries. However its performance for diffuse ischemic WMD detection is less effective than magnetic resonance imaging (MRI) and its predictive value for abnormal neurodevelopmental outcomes is not clearly established^7,8^.

Shear wave elastography (SWE) is an ultrasound modality able to measure the stiffness (i.e. Young’s elastic modulus) of a tissue^9^. Monitoring the evolution of brain stiffness in the neonatal period could help clinicians understand when WMD begins and how it evolves over time, as these lesions might be associated with a softening of brain tissue due to microglial activation and local inflammation of the white matter (WM)^10^. The principle of SWE relies on the measurement of the speed of a shear wave propagating in the tissue, as shear waves propagate faster in stiffer media. The shear wave is generated via the ultrasound beam and its propagation is imaged using ultrafast ultrasound imaging at several thousand frames per second^11^.

SWE provides a quantitative mapping of the stiffness (in kPa) within a tissue or organ with a contrast that will highlight areas of different stiffness, e.g. a hard inclusion, or a softer region. Several clinical applications have been developed for the detection of liver fibrosis or the detection of tumors in the breast, thyroid or kidney^12,13^.

Some studies have already shown the feasibility of SWE of the neonatal brain in a clinical context^14–16^. However, they only characterized normal values of elasticity within different brain regions or at different gestational ages, without any diagnostic or prognostic values, except for studies successfully linking elasticity measurements to increased intracranial pressure due to hydrocephalus^17,18^.

The aims of this study assessing 2-dimensional SWE were (i) to compare the stiffness of the neonatal brain between preterm and term infants and (ii) to assess its added-value to detect brain lesions in preterm infants early after birth compared to B-mode cUS and MRI at term equivalent age (TEA). We hypothesized that preterm newborns with WMD diagnosed on cUS or MRI would have lower elasticity values relative to other newborns.

## Patients and methods

### Patients

We conducted a prospective case–control study of newborns who were admitted to the Robert Debré Children’s Hospital, in Paris, France between January 2015 and April 2017. The determination of the sample size was not feasible when the protocol was written in 2013 due to the absence of comparable literature. Therefore, the number of participants was established empirically, considering the recruitment capacity of the neonatology unit within one year. We included preterm neonates born at a gestational age below 32 weeks and full-term control neonates born between 39^0/7^ and 40^6/7^ weeks. For secondary analyses, preterm infants were separated into two gestational age groups (24^0/7^ − 27^6/7^ weeks) for extreme preterm neonates and (28^0/7^ − 31^6/7^ weeks) for very preterm neonates. We excluded newborns with genetic or congenital abnormalities. The trial was approved by the national ethics committee (Comité de Protection des Personnes, Ile-de-France II, Necker), the French National Drug Safety Agency (EudraCT number 2012-A01530-43), and the French data protection authority (Commission Nationale de l'Informatique et des Libertés). Written informed consent was obtained from parents of all eligible infants before inclusion. The trial was first registered at ClinicalTrials.gov on 23/01/2014 under the number NCT02042716, before the first patient was enrolled, and complies with the ethical principles for medical research involving human subjects of the Declaration of Helsinki.

### Data collection

For each patient, clinical data referred to pregnancy (gestational diabetes, preeclampsia, antenatal steroids), birth (gestational age at birth, birth weight, sex, Apgar score at 5 min) and the presence of severe neonatal morbidities, including grade 3–4 intraventricular hemorrhage, cystic periventricular WMD, stage ≥ 2 necrotizing enterocolitis according to Bell’s staging, stage ≥ 3 retinopathy of prematurity according to international classification and/or laser treatment, and severe bronchopulmonary dysplasia at 36 weeks of postmenstrual age were prospectively recorded into the medical chart.

Infants were classified as small for gestational age if they were born with a birth weight < 10th percentile on customized AUDIPOG curves for male and female neonates^19^. Late onset sepsis was defined by the detection of a bacterial pathogen in the blood after 72 h of life requiring antibiotic treatment for more than 72 h^20^.

### Cranial ultrasound imaging (cUS)

All cUS were performed using the CE-marked Aixplorer ultrasound system (Supersonic Imagine, Aix-en-Provence, France) with a 6 MHz central frequency linear probe (SL10-2 Supersonic Imagine, 192 elements, pitch 0.2 mm). Both conventional B-mode cUS and SWE imaging sequences complies with international standard in terms of power and acoustic emission for pediatric transfontanellar ultrasound system applications (Food and Drug Administration (FDA) Track 3: (MI < 1.9, ISPTA < 720 mW/cm^2^ and ISPPA < 190 W/cm^2^). B-mode acquisitions were performed using sagittal and coronal planes, and Shear Wave Elastrography (SWE) mode were acquired using sagittal planes. The same three operators performed the examinations. The operators were radiology technologist specialized in paediatric ultrasound acquisition for more than 7 years. For preterm neonates, examinations were performed at day of life 3 (DOL 3), 8 (DOL 8), 21 (DOL 21) and at term equivalent age (TEA, 40 ± 1 weeks of postmenstrual age) in accordance with our standardized practice. For term neonates, there was only one acquisition at DOL 3.

#### Conventional cUS (B mode)

On sagittal and coronal planes, four cerebral regions were studied: left and right frontal WM, left and right parietal WM. For each region, 2 qualitative criterions were measured at each time point: the echogenicity compared to the choroid plexus (evaluated as hypoechoic, isoechoic or hyperechoic) and the global echogenicity (evaluated as homogenous or heterogenous). Examinations were classified by two paediatric radiologists with 5 and 20 years of experience, in consensus as normal if WM was hypoechoic compared to choroid plexus with a homogenous global echogenicity. When WM was iso or hyperechoic compared to choroid plexus or heterogeneous, it was considered as abnormal.

#### SWE imaging

The sagittal reference acquisition planes were selected on B-mode imaging. With SWE mode: real-time two-dimensional color-coded quantitative maps of brain stiffness (in kPa) were overlaid on the conventional gray-scale B-mode image. Measurements of brain stiffness were then performed by placing manually a region of interest in each of the following areas: left and right frontal WM, left and right parietal WM, left and right thalamus (see Supplementary Fig. S1).

For each patient and for each region, 3 different acquisitions with quantitative measurements were performed and the mean ± standard deviation (SD) of these 3 measurements were calculated and used for the statistical analyses.

SWE acquisitions were considered of good quality when the color-coded map was stable over several seconds and when the color-coded map was homogeneous, without major areas with non-measurable values.

#### Reproducibility of SWE measurement

For intra-observer reproducibility, acquisitions and measurements in the sagittal plane were repeated 3 times by the same observer during the same imaging session. For inter-observer reproducibility, two of the operators of the study, with 6–12 months of experience in SWE brain imaging, performed 3 acquisitions and measurements on the same day for each of the 10 randomly selected neonates. The mean stiffness value of the 3 acquisitions for each patient was calculated for each operator.

### Brain MRI scoring

All brain MRIs were performed without sedation on a 1.5 T Medical System with a standard head coil at TEA as previously described^21^.

MRI analysis and scoring was performed by two pediatric radiologists, with 5 and 20 years of experience, in consensus.

A standardized scoring system was used for evaluating cerebral WM, cortical and deep GM and cerebellum injuries of preterm neonates according to Kidokoro et al.^22^.

The occurrence of cerebral WMD was graded based on 6 variables: cystic lesion, focal signal abnormalities, delayed myelination, thinning of the corpus callosum, dilated lateral ventricles and reduction of WM volume. Total cerebral WMD score was reported as cumulative percentages and classified as normal-to-mild (0–4) and moderate-to-severe (≥ 5).

Cortical GM abnormality was graded based on 3 variables: signal abnormality, delayed gyration and increased extracerebral space.

Deep GM and cerebellum scores were graded based on signal abnormality and volume reduction.

An overall brain abnormality score was calculated as a sum of the four regional subscores and classified as normal-to-mild (0–7) and moderate-to-severe (≥ 8).

Presence of hemorrhagic lesions were analyzed according to Papile et al.^23^.

Diffusion weighted imaging was used to perform quantitative measurement of Apparent Diffusion Coefficient (ADC in ${10}^{-3} {{\text{mm}}}^{2}/{\text{s}}$) by manually placing area of interest in the same brain regions than SWE measurement: right and left frontal and parietal WM and right and left thalami. ADC value reflects microarchitecture by measuring diffusion movements of water molecules. ADC values decrease with brain maturation. ADC decrease in the acute phase of ischemic lesion but increase with chronic ischemic lesions. The relationship between elasticity and ADC values were studied.

### Statistical analysis

We performed statistical analysis on MATLAB software (Release 2021b, The MathWorks, Inc., Natick, Massachusetts, United States).

#### SWE reproducibility study

Intra and inter observers reproducibility studies were evaluated using a Bland–Altman test^24^ and concordance correlation coefficient (CCC)^25,26^. Reproducibility was classified as excellent (CCC ≥ 0.75), fair-to-good (CCC = 0.40–0.75) or poor (CCC ≤ 0.40)^27^. For Bland–Altman tests, the bias (mean difference) and 95% limits of agreement (confidence interval) are given.

#### Elasticity values comparison

To compare the elasticity values between term and preterm neonates, the cohort was separated in three groups: the first group of preterm neonates born between 24^0/7^ and 27^6/7^ weeks, the second group of preterm neonates born between 28^0/7^ and 32^6/7^ weeks and the third group of full-term neonates. For each region of interest (ROI), hemispheres were considered independent so a patient contributes two times (one for each hemisphere). Prospective measures of brain elasticity among preterm neonates were compared at DOL 3, DOL 8, DOL 21 and at TEA and to the control group of term neonates on DOL 3 using one-way analysis of variance (ANOVA), followed by post-hoc multiple comparisons using Tukey’s Honestly Significant Difference (HSD) test. To confirm the relationship between elasticity and age for each ROI, we performed a linear regression analysis of elasticity versus gestational age using the combined data from the two groups of preterm neonates.

The two groups of preterm children were pooled and then separated according to the WMD staging on conventional B-mode cUS as normal or abnormal in two regions of interest (ROI): the frontal WM and parietal WM. For each patient, both hemispheres of each ROI were studied independently at each measurement time, so that patients contributed 2 times to the analysis. To compare elasticity between the two groups, we first test the normality using Shapiro–Wilk test: if the data were normal, we performed a two-tailed Student’s t-test, otherwise a Wilcoxon rank sum test. After that a correction for multiple comparisons between days of acquisition was performed using the false discovery rate. For each statistical test a p-value less than 0.05 was considered significant. The same procedure was used to compare preterm neonates separated based on MRI classification of the WM as normal or abnormal. Results are presented as mean ± SD.

#### Relationship between elasticity values (SWE) and ADC values measured on term MRI

The relationship between elasticity values and ADC values were studied through a linear regression model.

## Results

### Patients

**Figure 1:**
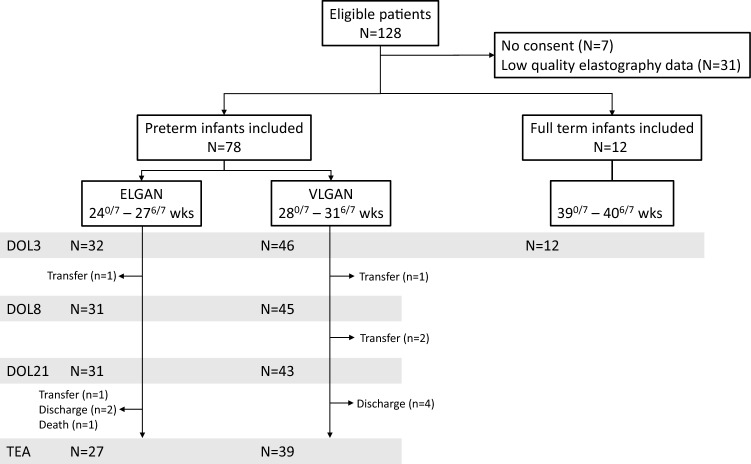
Patient flow diagram Abbreviations: ELGAN extremely low gestational neonate; VLGAN very low gestational neonate; DOL day of life; TEA term equivalent age.

Among 128 infants who met inclusion criteria, 38 patients were excluded including 7 without consent and 31 with insufficient SWE data quality. 90 infants were included, 32 extremely preterm infants born between 24^0/7^ and 27^6/7^ weeks, 46 very preterm infants born between 28^0/7^ and 31^6/7^ weeks, and 12 full-term infants (Fig. 1). At each time point of acquisitions (DOL 3, DOL 8, DOL 21 and TEA), the total number of patients in the preterm group varied due to discharge, hospital transfer or death. The main baseline characteristics for both gestational age groups are shown in Table 1. Cranial ultrasound findings (B mode) are available in Supplementary Table S1.

**Table 1:** Perinatal and neonatal characteristics according to the 3 gestational age groups.

*Variable*	24–27 weeks N = 32	28–31 weeks N = 46	39–40 weeks N = 12
Gestational age at birth in weeks, Median (IQR)	26.2 (25.7–27.1)	30.5 (29.3–30.9)	40.4 (39.8–40.7)
Male sex, no. (%)	14 (44)	21 (46)	8 (67)
Birthweight in grams, Median (IQR)	835 (725–923)	1300 (1100–1430)	3288 (3108–3785)
Small for gestational age (< 10th), no. (%)	2 (6)	6 (13)	2 (17)
Head circumference at birth in cm Median (IQR)	23 (22–24)	27 (26–28)	34.5 (34–35.5)
Multiple pregnancy, no. (%)	11 (34)	17 (37)	0 (0)
Antenatal steroids, no. (%)	29 (91)	42 (91)	0 (0)
Chorioamniotitis, no. (%)	19 (59)	12 (26)	0
Preeclampsia, no. (%)	5 (6)	15 (33)	0
Cesarean section, no. (%)	10 (31)	23 (50)	1 (8)
Apgar score at 5 min Median (IQR)	8 (6–10)	10 (9–10)	10 (10–10)
Exogenous surfactant for respiratory distress syndrome, no. (%)	18 (58)	15 (33)	0 (0)
Broncho-pulmonary dysplasia at 36 weeks PMA, no. (%)	10 (33)	0 (0)	0
Necrotizing enterocolitis stages II–III, no. (%)	0 (0)	1 (2)	0
Late onset sepsis, no. (%)	26 (81)	12 (26)	0
Retinopathy of prematurity, no. (%)	6 (19)	0 (0)	0
Intraventricular hemorrhage, no. (%)			
1–2 grade	11 (34)	10 (21)	0 (0)
3–4 grade	0 (0)	1(12)	0 (0)
Total white matter MRI score, no. (%)			
No (0–2)	14 (82)	5 (63)	
Mild (3–4)	3 (18)	2 (25)	
Moderate (5–6)	0 (0)	0 (0)	
Severe (≥ 7)	0 (0)	1 (12)	
Global abnormality MRI score, no. (%)			
No (0–3)	3 (18)	1 (13)	
Mild (4–7)	6 (35)	2 (25)	
Moderate (8–11)	2 (12)	1 (13)	
Severe (≥ 12)	6 (35)	4 (50)	

*IQR* interquartile range, *PMA* postmenstrual age, *MRI* magnetic resonance imaging.

### Intra and inter-observer reproducibility study for brain elasticity measurement with SWE

**Figure 2:**
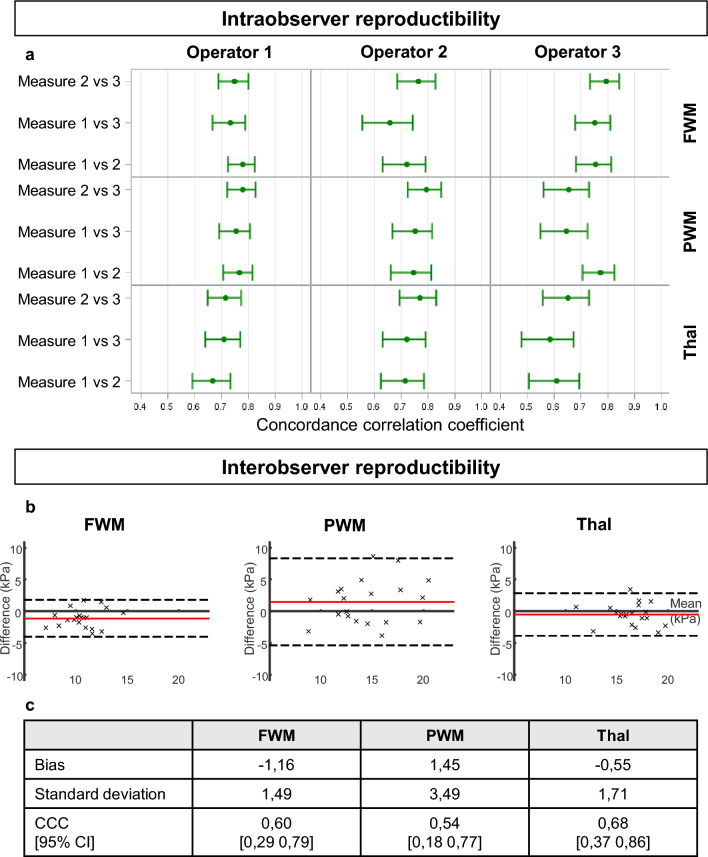
Inter- and intra-operators reproducibility. (**a**) Intra-operator reproducibility: for each operator and regions, the mean concordance correlation coefficient (CCC) was calculated over all available measurements. The green points represent the mean Concordance correlation coefficient (CCC) and the green bars represent the 95% confidence interval. (**b**) Inter-operator reproducibility: Bland Altman plot based on elasticity measurements performed by two operators on 10 neonates (randomly selected). The red line represents the bias or the mean difference between operators and the dotted lines represent the 95% confidence interval. (**c**) Values of bias, standard deviation and CCC for each brain regions. FWM = Frontal white matter; PWM = Parietal white matter; Thal = Thalamus.

As left and right hemispheres were considered independents for the measurements, a patient contributed two times. The concordance correlation coefficients (CCC) calculated for each of the 3 operators and each of the 3 regions measured showed a fair-to-good intra-observer reproducibility as they were all comprised between 0.4 and 0.75 (Fig. 2a) and the bias given by the Bland–Altman analysis were small (Supplementary Table S2). Frontal WM measurements were associated with the best intra-observer reproducibility.

The Bland–Altman plot showed also a good inter-observer reproducibility (Fig. 2b,c), as the CCC was superior to 0.4 for all regions, with a better reproducibility for frontal WM and the thalamus (CCC = 0.6 and CCC = 0.68) compared to the parietal WM (CCC = 0.54).

### Elasticity of the neonatal brain was dependent on gestational age at birth and brain areas

**Figure 3:**
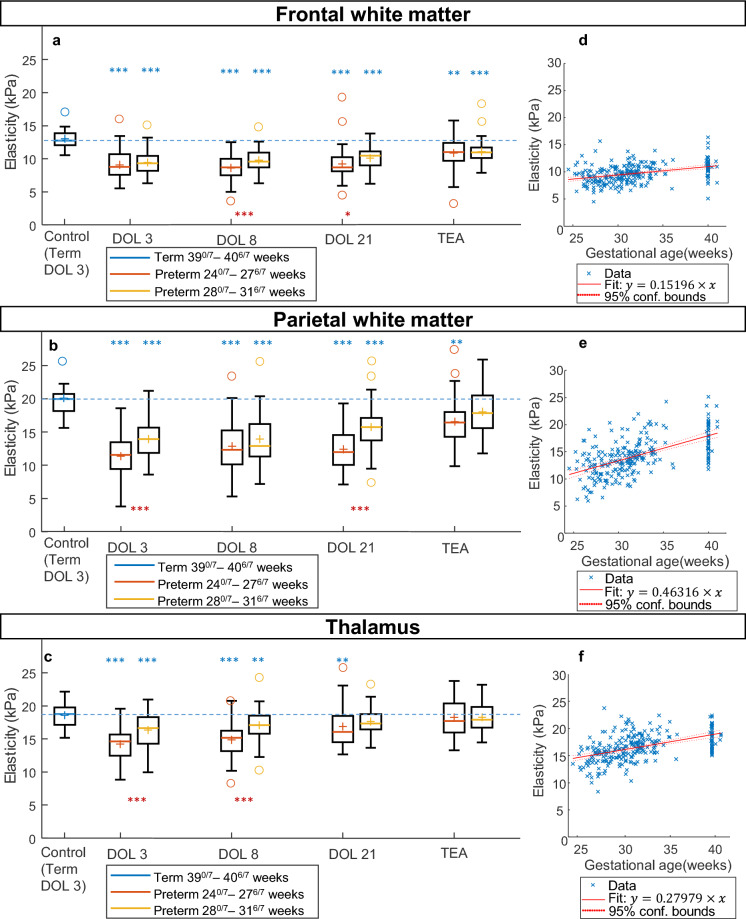
Evolution of the difference of the elasticity of brain regions between preterm and term neonates. (**a**–**c**) Frontal white matter (**a**), parietal white matter (**b**) and thalamus (**c**) elasticity values in preterm infants according to gestational age at birth (24^0/7^–27^6/7^ and 28^0/7^–31^6/7^ weeks) at each measurement time (DOL 3, DOL 8, DOL 21, TEA) compared to the control term newborn group (39^0/7^–40^6/7^ weeks) at DOL3. Blue stars represent significant differences with the term group (ANOVA), red stars represent significant differences between the two groups of preterm (ANOVA: ***p < 0.001, **p < 0.01, *p < 0.05). DOL day of life; TEA term equivalent age. (**d**–**f**) Linear model regression (red line) of Elasticity vs Gestational age in each brain regions for preterm neonates. For all regions, *p* < 0.001.

A comparison of brain elasticity between preterm and term neonates was performed at each time of acquisition in the frontal WM, parietal WM and thalami using one-way ANOVA (Supplementary Tables S3 and S4). We found that elasticity in the WM (parietal and frontal) was significantly lower in preterm infants than in term infants from birth (DOL 3) till TEA, *p* < 0.001 (Fig. 3a/b, blue stars). Overall, elasticity values were found to be lower in the extremely preterm infants compared to very preterm infants (Fig. 3a/b, red stars).

In the thalami, elasticity was also significantly lower in preterm compared to term infants from DOL 3 to DOL 21 (Fig. 3c, blue stars), but tended to reach at TEA the values measured in full-term controls. There was also a lower elasticity with lower gestational age at birth (Fig. 3c, red stars).

The linear regression slope (m) of elasticity values vs gestational for preterm neonates was positive in the frontal WM (m = 0.15, *p* < 0.001), the parietal WM (m = 0.46, *p* < 0.001) and the thalamus (m = 0.28, *p* < 0.001) (Fig. 3d–f). It confirmed that elasticity increased with gestational age. The raw data for each neonate's age-dependent elasticity is available in Supplementary Fig. S2 as a spaghetti plot.

### Elasticity of the frontal white matter was lower in neonates with abnormal echogenicity on conventional B-mode cUS

**Figure 4:**
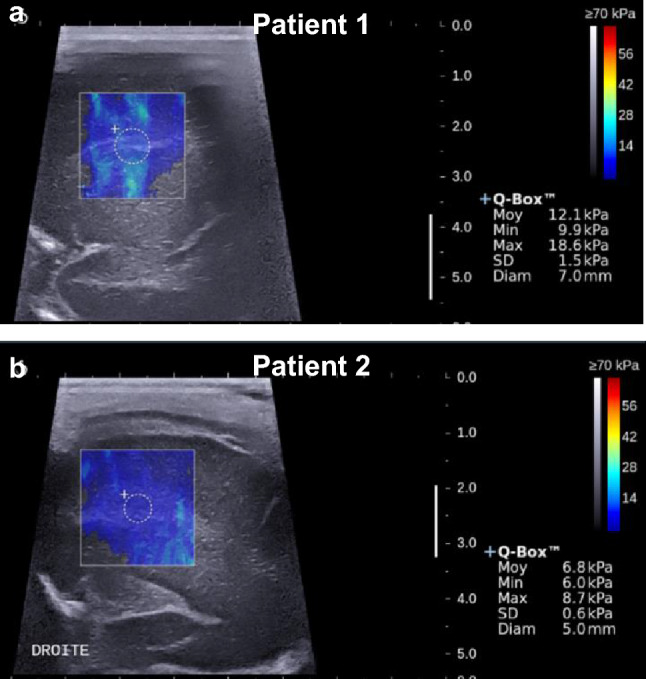
B-mode cranial ultrasound (cUS) images with overlaid shear wave elastography (SWE) measurements at DOL 3 in the right frontal white matter for two patients. B-mode cUS images are shown in grayscale and with overlaid SWE measurements in color-coded map for (**a**) Patient 1 with normal echogenicity on B-mode cUS image and (**b**) Patient 2 with abnormal echogenicity on the B-mode cUS image. The information in the Q-box is the summary of the SWE measurements. The corresponding mean elasticity values show that the elasticity is lower in the frontal white matter of Patient 2 with detected abnormal echogenicity (6.8 ± 2.7 kPa) compared to Patient 1 with normal echogenicity (12.1 ± 1.5 kPa).

Each infant was classified into normal or abnormal WM echogenicity using B-mode cUS at DOL3, DOL 8, and DOL 21 for frontal and parietal WM (see Supplementary Table S1). Figure 4 illustrates B-mode cUS and SWE measurements in the right frontal WM at DOL 3 with normal and abnormal WM echogenicity on B-mode cUS.

**Figure 5:**
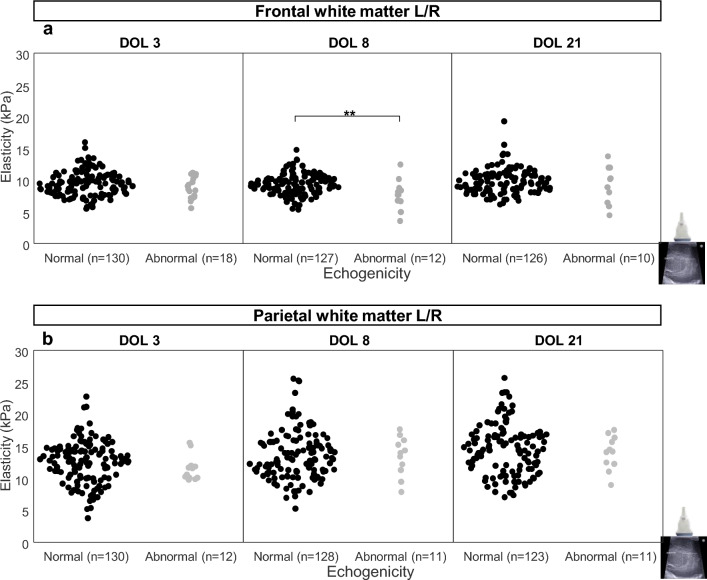
Comparison of white matter elasticity values measured on days 3, 8, 21 according to B-mode cUS abnormalities. (**a**) Elasticity in the left (L) and right (R) frontal white matter (**, *p* < 0.01). (**b**) Elasticity in the left (L) and right (R) parietal white matter. n represents the number of considered hemispheres as each patient contributes two times, one for each hemisphere) DOL: day of life.

The distributions of elasticity values according to the presence or absence of WM abnormalities on B-mode cUS are shown in Fig. 5. Values of elasticity of frontal WM were significantly lower in the abnormal WM group compared to the normal group on DOL 8 (7.83 ± 2.28 kPa vs. 9.40 ± 1.61 kPa, *p* < 0.01), but neither on DOL 3 (8.89 ± 1.71 kPa vs. 9.53 ± 1.97 kPa, *p* = 0.1949) nor on DOL 21 (9.22 ± 2.99 kPa vs. 9.81 ± 1.95 kPa, *p* = 0.6022) (Fig. 5a). Elasticity values of the parietal WM, whose variability was high, were found similar in infants with and without abnormal WM as assessed in that region by B-mode cUS (Fig. 5b).

### Elasticity of the frontal WM on DOL 3 to predict abnormal MRI at TEA infants born very preterm

**Figure 6:**
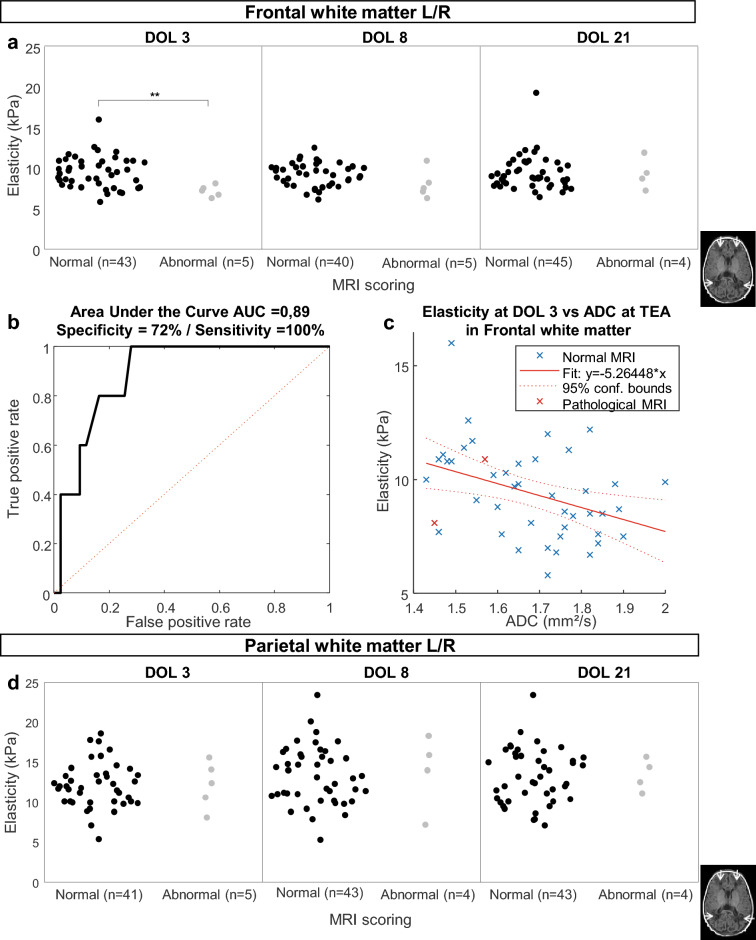
White matter elasticity comparison based on MRI classification (**a**) Comparison between elasticity values measured in the frontal white matter on day of life (DOL) 3, 8, 21 in preterm infants according to MRI scoring (normal vs abnormal) at term equivalent age (TEA) (n represents the number of considered hemispheres as each patient contributes two times, one for each hemisphere; **p < 0.01). (**b**) Receiver operating characteristic (ROC) curve analysis for frontal white matter lesion prediction based on elasticity values of the frontal white matter at DOL 3: (AUC of 0.89, max Youden index: sensitivity 100% and specificity 72% for a threshold of 8.4 kPa). (**c**) Linear regression of elasticity of frontal white matter on DOL 3 vs frontal apparent diffusion coefficient (ADC) from MRI at TEA in preterm infants. (**d**) Comparison between elasticity values measured in the parietal white matter on DOL 3, 8, 21 in preterm infants according to MRI scoring (normal vs abnormal) at TEA (n represents the number of considered hemispheres as each patient contributes two times, one for each hemisphere).

We next assessed whether early elasticity may predict subsequent MRI abnormalities at TEA. The subset of very preterm neonates assessed using MRI at TEA (n = 25) was classified as normal or abnormal for both frontal and parietal WM, according to Kidokoro score. The distribution of elasticity values for each group at each time point is depicted in Fig. 6a and d. Early elasticity values of the frontal WM were significantly lower in infants with abnormal MRI (WM Kidokoro score > 2) compared to those measured in infants with normal MRI (WM Kidokoro score ≤ 2) on DOL 3 (7.16 ± 0.70 kPa vs. 9.51 ± 1.95 kPa, *p* < 0.01). This difference was no longer observed on DOL 8 (8.00 ± 1.76 kPa vs. 9.20 ± 1.42 kPa, *p* = 0.09) and DOL 21 (9.30 ± 1.96 kPa vs. 9.37 ± 2.13 kPa, *p* = 0.98).

The discriminative value of early brain elasticity on DOL3 to predict frontal WMD detected on MRI at TEA was further assessed using a receiver operator characteristics (ROC) curve and showed an area under the curve (AUC) of 0.89 with a specificity of 72% and sensitivity of 100% (for a threshold of 8.4 kPa based on the maximum Youden index criteria) (Fig. 6b).

Elasticity and ADC values were negatively correlated at DOL 3 with slopes of − 5.26 kPa s/mm^2^ (*p* = 0.009) (Fig. 6c), a finding consistent with the ability of early elasticity to predict MRI findings at TEA in the frontal WM.

In contrast to frontal WM, no significant difference or correlation was observed between elasticity measured in parietal WM and MRI abnormalities (Fig. 6d).

## Discussion

In this study, we first described the developmental changes over time of brain elasticity measured by SWE within the WM and thalami in preterm neonates compared to full-term infants. We found higher elasticity in term neonates in agreement with previous studies^16^, and increasing elasticity values with gestational age at birth. Then, we showed that elasticity was lower for neonates whose WM was detected as damaged on B-mode cUS. Finally, we found that lower values of elasticity in the frontal WM at early postnatal age (DOL3) were a good predictor of WM damage as detected by MRI at TEA.

The interpretation of elasticity values and their changes with age and brain region can be directly related to the degree of myelination, as stiffness increases with myelin content^28^. Indeed, Weickenmeier et al.^28^ showed a high correlation between bovine brain myelin content (based on histological analysis) and stiffness (measured by force–displacement characterization in tissue samples). They hypothesized that the role of myelin is not limited to assisting the transmission of information between neurons but may also play a structural role in the brain folding and neurodevelopment by locally modulating the mechanical properties of the brain. This correlation between stiffness and myelin content was confirmed by a study showing that a controlled demyelination induced by cuprizone decreases the stiffness of the brain in mice using magnetic resonance elastography^29^. In humans, at a later age, magnetic resonance elastography has also shown a decrease in brain elasticity in patients with Alzheimer’s disease^30^ which is known to be linked to demyelination.

Regarding the estimation of the elasticity values in white and grey matters, studies in newborns have shown rather variable results^14–16,31^. Recording reliable measures of brain elasticity and studies comparison remain challenging for many reasons. First, different technologies are used by the ultrasound manufacturers for elasticity estimation. When comparing studies using the same ultrasound machine (either GE healthcare in^15,16^ or SuperSonic Imagine in^17^/our study), it seems that the values are more consistent. Also, WM mechanical anisotropy (induced by axons and myelin fibers local orientation) makes the velocity of shear waves vary depending on their direction of propagation: shear waves tends to propagate faster along the fibers than across the fibers^32^. Therefore, for a given anatomical location the apparent elasticity will be higher if measured along the fibers compared to across the fibers. The fibers orientation with regard to the shear wave propagation direction changes depending on the plane of acquisition (coronal or sagittal) and the measurement area (frontal or parietal) location and size. Standardization of the SWE measurement locations and imaging planes, as done in the present study, supports reproducibility of measurements. But some locations in the brain might have local complex anisotropy, or even strong inter-individual variability, that might increase the variance of elasticity measurements. This can probably explain the larger variability of elasticity values measured in the parietal WM compared to the frontal WM or thalamic measurements in our study and the worse interoperator-reproductibility for the parietal WM. For the frontal WM, the bias (mean difference) of interobserver reproducibility was larger than for the intra-observer analysis but still inferior to the observed differences between pathological and normal patients classified on MRI on DOL3. However, this bias is really close to the range of the difference between pathological and normal patients classified on B-mode cUS on DOL 8, so this result should be interpreted with caution.

It should also be noted that myelination is a continuous process, starting at 20 weeks of gestation, and progressing from posterior areas to anterior frontal WM, explaining why elasticity values of parietal WM are higher than frontal WM at any gestational age. It also means that the same region, measured at different gestational ages, will have a different degree of myelination and a different elasticity value.

Finally, as mentioned previously, the elasticity is linked to the molecular constitution of the medium, and the medium under consideration does not consist solely of myelin even in the WM. The vascular, neuronal and glial cells densities probably also have an effect on the measured elasticity. In particular, microglial cells populating the developing WM were reported to be frequently activated in the brain of preterm infants or in animal models of brain injury^33^. This glial activation and subsequent neuroinflammation can lead to abnormal microstructure and water content of the WM^34^. These data may explain how early elasticity in vulnerable periventricular WM areas may correlate with abnormal structural changes several weeks later on MRI. The potential clinical interest of SWE compared to B-Mode ultrasound or MRI lies both in 1/its ability to detect structural changes taking place upstream of the onset of WMD and in 2/its low-cost bedside use, allowing regular monitoring and increasing the chances of detecting abnormalities as early as possible.

As SWE uses the emission of an ultrasound beam to create a small shear deformation within the brain, the question of safety in terms of mechanical and thermal bioeffects on the neonatal developing brain is important. First, the induced displacement is small compared to the size of the focal spot, with a typical maximal displacement of 3 µm and a typical transverse width of the focal spot of 300 µm for the US frequency used (6 MHz). This results in a very low relative shear strain of 3/300 = 1%. Elastography has now been used for neonatal imaging in a consequent number of studies^14–18,35,36^ without any reported adverse effect, and some preclinical studies have found that SWE do not induce histological changes, behavioral changes or long term effect on memory^37,38^ even if a reversible and transient effect (< 24 h) on expression of mRNA involved in synaptic functions in the hippocampus was reported^38^.

The main strengths of our study include the granular, robust, and multimodal data collection, and the detailed longitudinal courses for all preterm infants. Indeed, B-mode cUS, SWE and MRI imaging were meticulously performed and reviewed by two authors to ensure consistency. Nevertheless, our study has some important limitations. Brain MRI was not performed in all preterm infants and accordingly, the severity and location of WM damage as well as their potential impact on neurodevelopmental outcomes were not evaluated. The study power is quite limited by the low incidence of WMD and the relatively small size of the extremely preterm group. Another limitation was the small number of neonates in the term control group and the lack of acquisitions on DOL 8 and DOL 21 due to the difficulty of recruiting term infants for extended periods as they typically only stayed in the maternity ward for a few days. Although we cannot compare with preterm measurements on the same timeline, this unique measurement allows us to compare the development of elasticity during the extra-uterine life of preterm neonates to a reference at birth with normal intra-uterine life development.

## Conclusion

This study shows that quantitative stiffness bedside measurement with 2D-SWE is both feasible and reproducible in preterm neonates. First, our results indicate that stiffness increases with gestational age which could be explained by the varying degree of myelination. Second, our findings also suggest that stiffness is lower in preterm infants with WMD before it is detected on MRI at TEA, allowing for good classification of preterm infants with WMD. However, further standardization of measurements is needed for improving reliable assessment across multi-site studies. Ultimately, we believe SWE imaging modality will allow clinically relevant improvements in the monitoring of the neonatal brain maturation from an early stage after birth and to predict WMD before TEA.

### Supplementary Information

Supplementary Information.

## Data Availability

Data and Code are available upon request to the corresponding author and will need formal data sharing agreement and approval from the requesting researcher's local ethics committee.

## Abbreviations

ADC: Apparent diffusion coefficient
BPD: Bronchopulmonary dysplasia
CCC: Concordance correlation coefficients
CI: Confidence interval
cUS: Cranial ultrasound
DOL: Day of life
IQR: Interquartile range
MRI: Magnetic resonance imaging
PDA: Patent ductus arteriosus
PMA: Postmenstrual age
SWE: Shear wave elastography
TEA: Term-equivalent age
WM: White matter
