# Supplementary material for: Transfontanellar shear wave elastography of the neonatal brain for quantitative evaluation of white matter damage
Source: Sci Rep. 2024 May 23;14:11827. doi: 10.1038/s41598-024-60968-w (PMC11116529; doi:10.1038/s41598-024-60968-w)
Supplement: Supplementary file 1 — Supplementary Information. [file 41598_2024_60968_MOESM1_ESM.pdf]

Transfontanellar shear wave elastography of the neonatal brain for quantitative evaluation of white matter damage

Flora Faure[1 +*], Marianne Alison[2 +], Mariantonietta Francavilla[3], Priscilla Boizeau[4], Sophie Guilmin Crepon[4], Chung Lim[2], Gregory Planchette[2], Mickael Prigent[2], Alice Frérot[5], Mickael Tanter[1], Charlie Demene[1], Olivier Baud[6 ++], Valérie Biran[5,7 ++*]

[1] Institute Physics for Medicine Paris, Inserm U1273, ESPCI Paris, PSL University, CNRS UMR 8063, 75015 Paris, France

[2] Assistance Publique-Hôpitaux de Paris, Pediatric Radiology Department, Robert Debré University Hospital, 75019 Paris, France.

[3] A.O.U.C. Policlinico of Bari - Hospital Giovanni XXIII, Pediatric Radiology Department, Bari, Italy

[4] Assistance Publique-Hôpitaux de Paris, Unit of Clinical Epidemiology, Robert Debré children's hospital, University of Paris Cité, Inserm U1123 and CIC-EC 1426, Paris, France.

[5]Assistance Publique-Hôpitaux de Paris, Neonatal intensive care unit, Robert Debré children's hospital, Paris, France.

[6] Division of Neonatology and Paediatric Intensive Care, Children's University Hospital of Geneva and University of Geneva, Geneva, Switzerland.

[7] Inserm U1141, University of Paris Cité, Paris, France.

[+] Flora Faure and Marianne Alison are co-first authors.

[++] Olivier Baud and Valérie Biran are co-last authors.

Email addresses:

F.F : flora.faure@hotmail.fr
M.A. : marianne.alison@aphp.fr
M.F. : marianto_fra@hotmail.it
P.B. : priscilla.boizeau@aphp.fr
S.G-C : sophie.guilmin-crepon@aphp.fr
C.L. : chungums@yahoo.fr
G.P. : gregory.planchette@aphp.fr
M.P. : mickael.prigent@aphp.fr
A.F. : alice.frerot@aphp.fr
M.T. : mickael.tanter@espci.fr
C.D. : charlie.demene@espci.fr
O.B. : olivier.baud@hcuge.ch
V.B.: valerie.biran@gmail.com

Correspondance to:

Prof. Valérie BIRAN, Department of Neonatal Intensive Care Unit, Assistance Publique-Hôpitaux de Paris, Robert Debré children's hospital, Paris, France.

Tel : +33 140034191 Email: valerie.biran@gmail.com

ORCID: 0000-0002-0284-3103

Supplementary Information

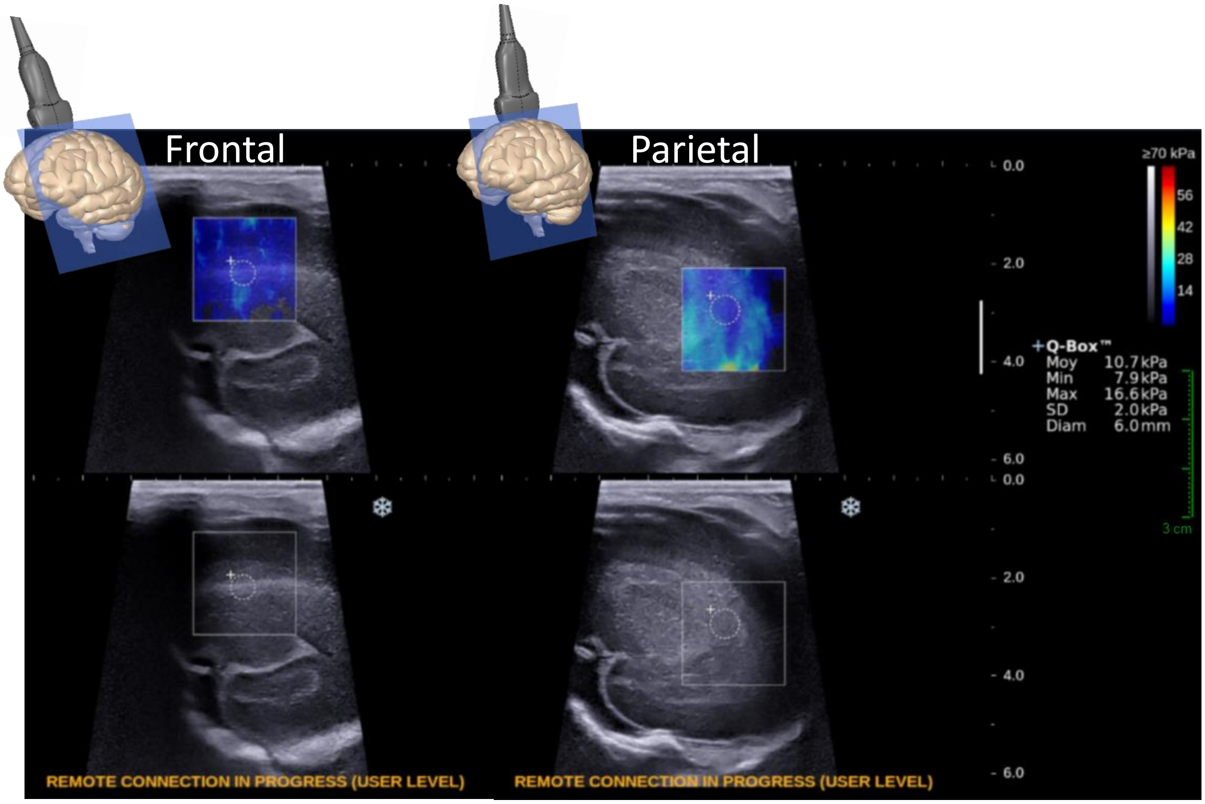

Supplementary Figure S1. Ultrasound imaging modalities in frontal and parietal areas on a patient on day 3 of life (DOL 3) from sagittal planes over the anterior fontanelle. (Up)B-mode imaging with superimposed shear wave elastography cartography. The measure is the mean elasticity within the dotted circle. (Down) B-mode imaging with conventional ultrasound examination.

Supplementary Table S1. Cranial ultrasound findings (B mode) in population subgroups.

Echogenicity<br>Expressed as count (percentage)	24-27 weeks	28-31 weeks	39-40 weeks	MD
**DOL 3**				
	N=32	N=46	N=12	
**Right frontal WM**<br>**Normal**<br>**Pathological**	N=32<br>27 (84%)<br>5 (16%)	N=46<br>41 (89%)<br>5 (11%)	N=12<br>12 (100%)<br>0 (0%)	0/0/0
**Left frontal WM**<br>Normal<br>Pathological	N=32<br>25 (78%)<br>7 (22%)	N=46<br>43 (93%)<br>3 (7%)	N=12<br>12 (100%)<br>0 (0%)	0/0/0
**Right posterior periventricular WM**<br>Normal<br>Pathological	N=32<br>30 (94%)<br>2 (6%)	N=46<br>43 (93%)<br>3 (7%)	N=12<br>12 (100%)<br>0 (0%)	0/0/0
**Left posterior periventricular WM**<br>Normal<br>Pathological	N=32<br>29 (91%)<br>3 (9%)	N=46<br>42 (91%)<br>4 (9%)	N=12<br>12 (100%)<br>0 (0%)	0/0/0
**DOL 8**				
	N=31	N=45		
**Right frontal WM**<br>Normal<br>Pathological	N=30<br>26 (87%)<br>4 (13%)	N=41<br>40 (98%)<br>1 (2%)		1/4
**Left frontal WM**<br>Normal<br>Pathological	N=30<br>24 (80%)<br>6 (20%)	N=41<br>40 (98%)<br>1 (2%)		1/4
**Right posterior periventricular WM**<br>Normal<br>Pathological	N=30<br>27 (90%)<br>3 (10%)	N=41<br>39 (95%)<br>2 (5%)		1/4
**Left posterior periventricular WM**<br>Normal<br>Pathological	N=30<br>27 (90%)<br>3 (10%)	N=41<br>38 (93%)<br>3 (7%)		1/4
**DOL 21**				
	N=31	N=43		
**Right frontal WM**<br>Normal<br>Pathological	N=28<br>26 (93%)<br>2 (7%)	N=41<br>39 (95%)<br>2 (5%)		3/2

Echogenicity<br>Expressed as count (percentage)	24-27 weeks	28-31 weeks	39-40 weeks	MD
**Left frontal WM**<br>Normal<br>Pathological	N=28<br>24 (86%)<br>4 (14%)	N=41<br>39 (95%)<br>2 (5%)		3/2
**Right posterior periventricular WM**<br>Normal<br>Pathological	N=28<br>26 (93%)<br>2 (7%)	N=41<br>37 (90%)<br>4 (10%)		3/2
**Left posterior periventricular WM**<br>**Normal**<br>**Pathological**	N=28<br>26 (93%)<br>2 (7%)	N=41<br>38 (93%)<br>3 (7%)		3/2
**TEA**				
	N=27	N=39		
**Right frontal WM**<br>**Normal**<br>**Pathological**	N=18<br>16 (89%)<br>2 (11%)	N=31<br>30 (97%)<br>1 (3%)		9/8
**Left frontal WM**<br>**Normal**<br>**Pathological**	N=17<br>15 (88%)<br>2 (12%)	N=31<br>31 (100%)<br>0 (0%)		10/8
**Right posterior periventricular WM**<br>**Normal**<br>**Pathological**	N=18<br>17 (94%)<br>1 (6%)	N=31<br>31 (100%)<br>0 (0%)		9/8
**Left posterior periventricular WM**<br>**Normal**<br>**Pathological**	N=18<br>18 (100%)<br>0 (0%)	N=31<br>31 (100%)<br>0 (0%)		9/8

Abbreviations: MD: missing data; WM: white matter; DOL: day of life; TEA: term equivalent age.

Supplementary Table S2. Intra-observer reproducibility of measurements

	N	Mean difference [CI 95%]	Concordance coefficient [CI 95%]
**Operator 1**			
**Thalamus in kPa**			
Measures 1 vs 2	232	0.11 [-5.2;5.42]	0.67 [0.59;0.73]
Measures 1 vs 3	232	-0.24 [-5.28;4.8]	0.71 [0.64;0.77]
Measures 2 vs 3	232	-0.35 [-5.12;4.42]	0.72 [0.65;0.77]
**Frontal white matter in kPa**			
Measures 1 vs 2	232	-0.01 [-3;2.98]	0.78 [0.72;0.83]
Measures 1 vs 3	230	-0.07 [-3.33;3.19]	0.73 [0.67;0.79]
Measures 2 vs 3	230	-0.05 [-3.14;3.05]	0.75 [0.69;0.8]
**Parietal white matter in kPa**			
Measures 1 vs 2	223	-0.17 [-6.34;6]	0.77 [0.71;0.82]
Measures 1 vs 3	221	0.14 [-6.15;6.43]	0.75 [0.69;0.81]
Measures 2 vs 3	221	0.29 [-5.72;6.3]	0.78 [0.72;0.83]
**Operator 2**			
**Thalamus in kPa**			
Measures 1 vs 2	142	0.11 [-4.29;4.51]	0.71 [0.62;0.79]
Measures 1 vs 3	141	0.01 [-4.09;4.12]	0.72 [0.63;0.79]
Mesures 2 vs 3	141	-0.14 [-3.83;3.54]	0.77 [0.69;0.83]
**Frontal white matter in kPa**			
Measures 1 vs 2	141	-0.06 [-3.94;3.82]	0.72 [0.63;0.79]
Measures 1 vs 3	139	0.02 [-4.16;4.2]	0.66 [0.55;0.74]
Measures 2 vs 3	139	0.08 [-3.5;3.65]	0.76 [0.69;0.83]
**Parietal white matter in kPa**			
Measures 1 vs 2	138	0.13 [-7.03;7.29]	0.75 [0.66;0.81]
Measures 1 vs 3	136	-0.27 [-6.76;6.22]	0.75 [0.67;0.82]
Measures 2 vs 3	136	-0.39 [-6.3;5.53]	0.79 [0.72;0.85]
**Operator 3**			
**Thalamus in kPa**			
Measures 1 vs 2	176	-0.25 [-5.65;5.15]	0.61 [0.51;0.69]
Measures 1 vs 3	176	-0.21 [-5.5;5.09]	0.58 [0.48;0.67]
Measures 2 vs 3	176	0.04 [-4.95;5.04]	0.65 [0.56;0.73]
**Frontal white matter in kPa**			

Measures 1 vs 2	176	0.31 [-2.6;3.22]	0.75 [0.68;0.81]
Measures 1 vs 3	176	0.41 [-2.37;3.2]	0.75 [0.68;0.81]
Measures 2 vs 3	176	0.10 [-2.53;2.73]	0.79 [0.73;0.84]
**Parietal white matter in kPa**			
Measures 1 vs 2	176	-0.23 [-5;4.53]	0.77 [0.7;0.83]
Measures 1 vs 3	176	-0.66 [-6.66;5.34]	0.64 [0.55;0.72]
Measures 2 vs 3	176	-0.42 [-6.33;5.48]	0.65 [0.56;0.73]

Abbreviations: CI : Confidence interval

Supplementary Table S3. White matter and thalamus elasticity values (expressed in kPa) in population subgroups.

Elasticity<br>Expressed as median (IQR)	24-27 weeks	28-31 weeks	39-40 weeks	MD
**DOL 3**				
	**N=32**	**N=46**	**N=12**	
**Average of frontal WM values R and L**	8.9 (7.8-9.8)	9.7 (8.7-10.5)	12.8 (11.8-13.5)	0/5/0
**Average of parietal WM values R and L**	11.9 (9.2-13.6)	13.5 (11.9-14.7)	20.0 (18.7-22.3)	3/7/2
**Average of thalamus values R and L**	14.1 (12.8-15.8)	16.6 (14.7-17.6)	18.9 (17.6-20.1)	0/4/0
**DOL 8**				
	**N=31**	**N=45**		
**Average of frontal WM values R and L**	8.5 (7.6-10.0)	9.6 (8.9-10.7)		3/5
**Average of parietal WM values R and L**	12.6 (11.0-15.0)	13.5 (12.1-16.1)		1/7
**Average of thalamus values R and L**	15.3 (13.9-16.3)	16.9 (15.7-18.5)		1/5
**DOL 21**				
	**N=31**	**N=43**		
**Average of frontal WM values R and L**	9.0 (8.2-10.2)	10.3 (9.2-11.5)		4/3
**Average of parietal WM values R and L**	11.7 (10.1-14.3)	15.2 (14.2-17.2)		7/2
**Average of thalamus values R and L**	16.3 (15.2-18.2)	17.4 (16.0-18.8)		4/2
**TEA**				
	**N=27**	**N=39**		
**Average of frontal WM values R and L**	11.1 (10.3-12.0)	10.9 (10.2-11.8)		9/8
**Average of parietal WM values R and L**	16.7 (14.1-18.4)	18.7 (15.9-20.1)		10/8
**Average of thalamus values R and L**	18.7 (16.0-20.2)	18.0 (16.7-19.6)		9/8

Abbreviations: MD: missing data; WM: white matter; R: right; L: left; DOL: Day of life; TEA: term age equivalent; IQR: Interquartile range

Supplementary Table S4. Comparison of elasticity of frontal white matter, parietal white matter and thalamus between the two groups of preterm infants (24-27 weeks and 28-31 weeks) and the control group (39-40 weeks).

DOL 3

Measurement	Groups	Estimation β	CI 95%	P-value
**Frontal white matter in kPa**	39-40 weeks	0	-	-
	24-27 weeks	-3.75	[-4.83 ;-2.67]	**p<0.001**
	28-31 weeks	-3.10	[-4.14 ;-2.05]	**p<0.001**
	24-27 vs 28-31	-0.65	[-1.40;0.09]	0.10
**Parietal white matter in kPa**	39-40 weeks	0	-	-
	24-27 weeks	-8.58	[-10.39; -6.77]	**p<0.001**
	28-31 weeks	-6.53	[-8.27;- 4.78]	**p<0.001**
	24-27 vs 28-31	-2.05	[-3.27;-0.84]	**0.0002**
**Thalamus in kPa**	39-40 weeks	0	-	-
	24-27 weeks	-4.56	[-6.00 ;-3.12]	**p<0.001**
	28-31 weeks	-2.55	[-3.94 ;-1.16]	**p<0.001**
	24-27 vs 28-31	-2.01	[-3.01;-1.02]	**p<0.001**

DOL 8

Measurement	Groups	Estimation β	CI 95%	P-value
**Frontal white matter in kPa**	39-40 weeks	0	-	-
	24-27 weeks	-4.24	[-5.18 ;-3.28]	**p<0.001**
	28-31 weeks	-3.07	[-3.98 ;-2.16]	**p<0.001**
	24-27 vs 28-31	-0.95	[-1.84;-0.49]	**0.0002**
**Parietal white matter in kPa**	39-40 weeks	0	-	-
	24-27 weeks	-7.03	[-9.14 ;-4.73]	**p<0.001**
	28-31 weeks	-5.60	[-7.64 ;-3.56]	**p<0.001**
	24-27 vs 28-31	-1.43	[-2.86;-0.01]	0.072
**Thalamus in kPa**	39-40 weeks	0	-	-
	24-27 weeks	-3.94	[-5.37 ;-2.52]	**p<0.001**
	28-31 weeks	-1.87	[-3.24 ;-0.50]	**0.004**
	24-27 vs 28-31	-2.07	[-3.07;-1.06]	**p<0.001**

DOL 21

Measurement	Groups	Estimation β	CI 95%	P-value
**Frontal white matter in kPa**	39-40 weeks	0	-	-
	24-27 weeks	-3.64	[-4.77 ;-2.50]	**p<0.001**
	28-31 weeks	-2.69	[-3.77;-1.61]	**p<0.001**
	24-27 vs 28-31	-0.95	[-1.76;-0.13]	**0.017**
**Parietal white matter in kPa**	39-40 weeks	0	-	-
	24-27 weeks	-7.45	[-9.52;-5.38]	**p<0.001**
	28-31 weeks	-4.05	[-6.00 ;-2.11]	**p<0.001**
	24-27 vs 28-31	-3.39	[-4.82;-1.97]	**p<0.001**
**Thalamus in kPa**	39-40 weeks	0	-	-
	24-27 weeks	-1.91	[-3.41 ;-0.41]	**0.008**
	28-31 weeks	-1.29	[--2.71 ;0.13]	0.08
	24-27 vs 28-31	-0.62	[-1.68;0.45]	0.37

TEA

Measurement	Groups	Estimation β	CI 95%	P-value
**Frontal white matter in kPa**	39-40 weeks	0	-	-
	24-27 weeks	-1.96	[-3.24; -0.68]	**0.0012**
	28-31 weeks	- 1.94	[-3.10; -0.76]	**0.0004**
	24-27 vs 28-31	-0.03	[-1.04;0.99]	0.99
**Parietal white matter in kPa**	39-40 weeks	0	-	-
	24-27 weeks	-3.31	[-5.47;-1.15]	**0.0012**
	28-31 weeks	-1.67	-3.64;0.30]	0.1145
	24-27 vs 28-31	-1.64	[-3.29;0.01]	0.0520
**Thalamus in kPa**	39-40 weeks	0	-	-
	24-27 weeks	-0.52	[-2.04 ;1.00]	0.69
	28-31 weeks	-0.51	[-1.89 ;0.88]	0.66
	24-27 vs 28-31	-0.01	[-1.22 ;1.19]	0.99

Abbreviations: CI: confidence interval; TEA: term equivalent age.

*Odd ratio and 95% confidence interval of Wald.

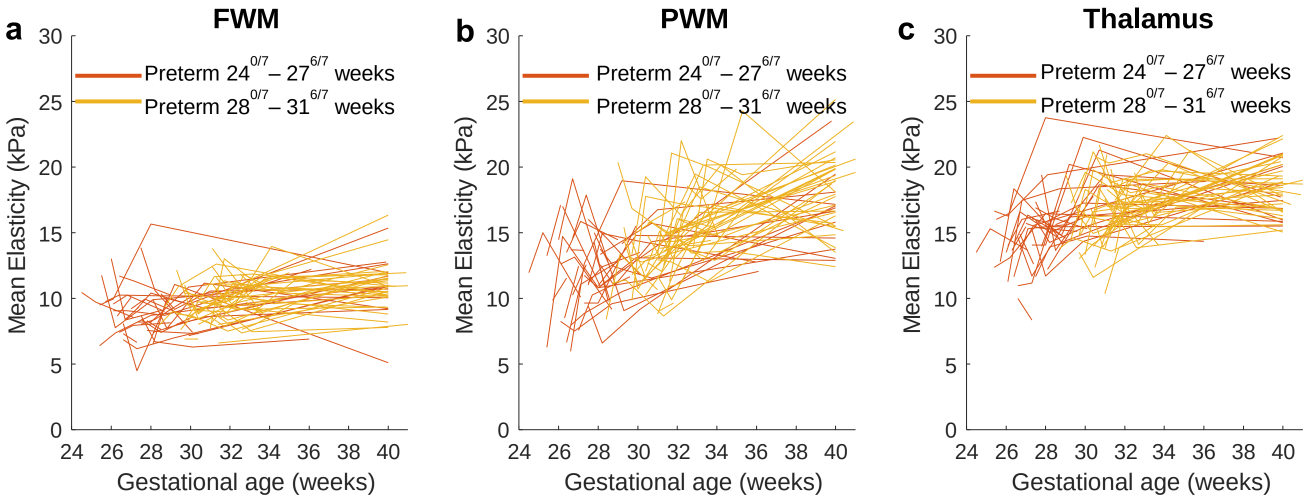

Supplementary Figure S2. Spaghetti plot of elasticity over time in 3 regions of interest: (a) Frontal white matter (FWM), (b) Parietal white matter (PWM) and (c) Thalamus. Each colored line represents raw measurements for a preterm neonate (yellow line if born between $24^{0/7}$ and $27^{6/7}$, orange line if born between $28^{0/7}$ and $31^{6/7}$).
